# Leptin/adiponectin ratio as a prognostic factor for increased weight gain in girls with central precocious puberty

**DOI:** 10.3389/fendo.2023.1101399

**Published:** 2023-03-10

**Authors:** Jessie Nallely Zurita-Cruz, Miguel Angel Villasís-Keever, Leticia Manuel-Apolinar, Leticia Damasio-Santana, Eulalia Garrido-Magaña, Aleida de Jesús Rivera-Hernández

**Affiliations:** ^1^ Medicine Faculty of Autonomous National University, Clinical Research Department, Hospital Infantil de México Federico Gómez, Ciudad de Mexico, Mexico; ^2^ Unit of Analysis and Synthesis of the Evidence, National Medical Center XXI Century, Instituto Mexicano del Seguro Social, Ciudad de Mexico, Mexico; ^3^ Department of Endocrinology Research, Hospital of Medical Specialties, National Medical Center XXI Century, Instituto Mexicano del Seguro Social, Ciudad de Mexico, Mexico; ^4^ Department of Pediatric Endocrinology, Children’s Hospital, National Medical Center XXI Century, Instituto Mexicano del Seguro Social, Ciudad de Mexico, Mexico

**Keywords:** leptin, adiponectin, precocious puberty, obesity, nutrition status, weight gain, prognosis

## Abstract

**Objective:**

To determine if the leptin, adiponectin, and leptin/adiponectin ratio (LAR) can predict weight gain at the end of GnRH analogs (GnRHa) treatment in girls with central precocious puberty (CPP).

**Material and methods:**

Study design: prospective cohort. Serum levels of leptin and adiponectin were determined at diagnosis of CPP. Anthropometry was performed at diagnosis of CPP and every six-months, until treatment with GnRHa was discontinued and they presented menarche. Patients were divided according to BMI<94 and BMI>95 percentile at diagnosis of CPP. The outcome was the increased in weight gain (e.g., from normal weight to overweight) at the end of follow-up. Statistical analysis: repeated measures ANOVA test and Student’s t-test were used to compare groups. Logistic regression analysis was used to evaluate the association of leptin and adiponectin levels, as well as LAR values with increased weight gain.

**Results:**

Fifty-six CPP patients were studied, 18 had BMI >95 percentile and 38 BMI <94 percentile. Of the 18 patients who initially had BMI >95^th^, two patients went from obesity to overweight, while among the 38 patients who started with BMI <94^th^, 21 (55.2%) increased their weight gain at the end of follow-up. This last group had higher leptin levels (8.99 ± 0.6 *vs* 6.14 ± 0.8, p=0.005) and higher LAR values compared to those who remained in the same weight (1.3 ± 0.5 *vs* 0.96 ± 0.56, p=0.01). In the logistic regression analysis, it was found that higher leptin levels and higher LAR values were associated with increased weight gain (RR 1.31, 95%CI 1.03-1.66, RR 4.86, 95%CI 1.10-21.51, respectively), regardless of birth weight, pubertal stage, age, and bone/chronological age ratio.

**Conclusions:**

In patients with CPP, leptin levels and higher LAR values appear to be associated with significantly greater weight gain during GhRHa treatment, particularly in girls starting with BMI < 94 percentile.

## Introduction

Central precocious puberty (CPP) is defined as the development of sexual characteristics before 9 years of age in boys and 8 years of age in girls due to activation of the hypothalamic-pituitary-gonadal axis (1, 2). CPP is a rare disease, with an estimated incidence of 1:10,000, but predominantly occurs in girls in more than 95% of cases (3, 4). There are many theories regarding the etiology of idiopathic CPP. One of which points out that these girls have a higher percentage of body fat for their chronological age, where leptin has a main role (5). Leptin acts through its receptor to stimulate the secretion of kisspeptin, a hypothalamic hormone, which in turn promotes the secretion of GnRH in the arcuate nucleus (6, 7).

It is common for girls with CPP to be overweight or obese at the time of diagnosis, but there are conflicting results as to whether weight is increased during or after receiving treatment with GnRH analogs (GnRHa), because there are studies indicating that the BMI increases and in others that it decreases (8–13). However, CPP is considered to be a risk factor for the development of cardiometabolic disorders in adult life, regardless of the presence of obesity (14, 15).

Adipose tissue is considered an endocrine organ that produces a wide variety of biologically active adipokines, such as leptin, adiponectin, tumor necrosis factor-α and interleukin-6 (16).

These adipokines plays a pathophysiological link between dysfunctional adipose tissue and cardiometabolic factors (17). Leptin is produced proportionally to the amount of body fat, and in patients with obesity there is an association of high leptin levels with cardiometabolic factors and metabolic syndrome (18, 19), while high levels of adiponectin are associated with a better metabolic profile in these patients (20). However, it has been observed that the leptin/adiponectin ratio (LAR) identify dysfunctional adipose tissue more accurately (21, 22). Thus, higher LAR values have been considered a better marker of insulin resistance and metabolic syndrome than serum leptin or adiponectin levels (23). Although the possible role of LAR in girls with CPP has not been studied, it is hypothesized that higher LAR values are a better predictor of weight gain than high leptin levels or low adiponectin levels.

The aim of this study was to determine if the leptin, adiponectin levels, and LAR can predict weight gain at the end of GnRha treatment in girls with CPP.

## Material and methods

### Subjects

A prospective cohort study was performed from January 2012 to May 2019 at a tertiary care pediatric center in Mexico City. For 36 months, we followed a cohort of Mexican girls <8 years of age with CPP. All included patients were selected using a consecutive sampling technique. At the time of diagnosis, subjects were classified as Tanner stages II and III. Follow-up started at the time of CPP diagnosis and initiation of leuprolide treatment. We excluded patients with any other disease or therapy associated with weight gain or increased appetite, such as intracranial tumors, Cushing disease, genetic syndromes (e.g., Prader-Willi, Bardet-Biedl, or Alstrom), use of steroids, fluoxetine, insulin sensitizers, hyporexigens, growth hormone, intestinal fat absorption inhibitors, or low birthweight (24).

We found 72 patients who fulfilled the inclusion criteria; but nine patients were excluded: three due to congenital adrenal hyperplasia, four due to low birthweight, and two because the parents did not agree to their daughter participating in the study. Finally, seven patients were eliminated because they were lost to follow-up. Thus, 56 patients were analyzed.

Leuprolide (GnRha) treatment consisted of monthly intramuscular application from diagnosis, discontinuing when patients reached a chronological age of 11 to 12 years, bone age 13 years or older, and growth velocity slowed to <4 cm/year (25–27). Subsequently, the patients continued to be monitored, until they presented menarche, which was the final moment of follow-up.

The research protocol was approved by the hospital ethics and research committee. Parents signed the informed consent and child assent according to the recommendations of the Declaration of Helsinki.

### Definitions

Diagnosis of CPP was made according to the following clinical criteria: Tanner breast stage II or higher, height acceleration, advancement of bone age (28) and confirmed by GnRHa stimulation test. This test is carried out with the application of 3.75 of GnRHa (leuprolide) and, after two hours, luteinizing hormone (LH) levels are measured (29). CPP is diagnosed with LH levels >7 mU/ml (29, 30). Adequate suppression of pituitary−gonadal function was defined as a stimulated plasma LH level after GnRH stimulation <6.5 mU/ml at 3, 12, 24 and 36 months, after treatment initiation with GnRHa (29).

Increase in weight gain and in BMIz, until the last evaluation were the primary outcomes measures. A BMI <84 percentile was considered normal weight, while a BMI between 84 and 95 percentile was considered overweight, and obesity when BMI was >95 percentile (31).

### Serum leptin and adiponectin measurements

Twelve-hour fasting serum leptin levels were measured between 7:00 and 8:00 a.m. using venipuncture samples at study onset and at a 12-month follow-up. Plasma samples were frozen at -20°C and analyzed. Leptin and adiponectin levels were measured using an enzyme-linked immunosorbent assay (ELISA) (Human Leptin DuoSet DY 398, R&D Systems, Minneapolis, MN, USA) (Human Adiponectin DuoSet DY 1065, R&D Systems, Minneapolis, MN, USA). All ELISA experiments were determined using Finstruments Multiskan EX (MTX Lab Systems Inc., Vienna, VA, USA) in duplicate per the manufacturer’s recommendations. The intra- and inter-assay coefficients of variation for all measurements were <7%. A standard curve was also included within each assay. The LAR was obtained by dividing the serum concentrations of leptin by those of adiponectin.

### Anthropometry

The patients’ anthropometric measurements were noted by a certified nutritionist and included height, weight, and body fat percentage by bioimpedance (Tanita BC-568 segmental analyzer, Tokyo, Japan). These anthropometric data were assessed every six months until the end of the follow-up.

### Statistics analyses

The Shapiro-Wilk test was applied to the quantitative variables, and a nonparametric distribution was observed. The quantitative variables were normalized by taking the logarithmic, except LAR distribution was normalized by taking the square root. We calculated the mean and standard error (SE) of quantitative variables.

Since patients with normal weight and overweight had increased weight gain compared to those with obesity, at the end of follow-up, two groups were formed according to the baseline BMI percentile (<94 and >95), to carry out all the analyses.

To determine differences in BMIz at study onset and after 12, 24 months, end of treatment and menarche, statistical analysis was performed using repeated measures ANOVA test and Student’s t-test to compare groups.

A logistic regression analysis was used to determine the association of LAR with increased weight gain, adjusted by birthweight, pubertal stage, age, and bone/chronological age ratio.

A p value <0.05 was considered statistically significant. STATA v.14.0 was used for all statistical analyses.

## Results

### Baseline

**Figure 1:**
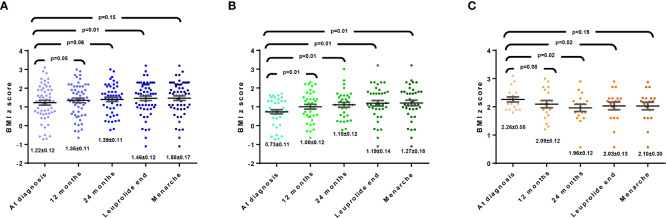
**(A)** Modification of BMI Z-score in all girls with CCP. **(B)** Modification of BMI Z-score in girls with BMI<94 percentile at diagnosis. **(C)** Modification of BMI Z-score in girls with BMI>95 percentile at diagnosis.

At diagnosis, the mean age of the 56 patients was 7.0 ± 0.18 years, mean bone age was 10.0 ± 0.26 years, and all patients had a bone/chronological age ratio >1. Of the total, 33 patients (58.9%) had puberty onset in Tanner breast stage II, and 23 patients (41.1%) were in breast stage III (see **Table 1**). The BMIz mean was 1.22 ± 0.12; 25 patients (44.6%) had normal BMIz, 13 patients (23.2%) had overweight, and 18 patients (32.1%) had obesity (**Table 1** and **Figure 1**).

**Table 1:** Baseline characteristics of 56 girls with central precocious puberty, according to the BMI at the beginning of follow-up.

		All patients	BMI <94 percentile	BMI >95 percentile
		n=56	n=38	n=18
		Mean ± SE		
Chronological age, years		7.0 ± 0.18	6.8 ± 0.24	7.4 ± 0.18
Bone age, years		10.0 ± 0.26	9.6± 0.35	10.9 ± 0.26
Birth weight, kg		2.9 ± 0.56	2.8 ± 0.73	3.0 ± 0.79
BMI z-score		1.22 ± 0.12	0.73 ± 0.11	2.26 ± 0.08
Stratification according to BMI percentiles	Normal	25 (44.6)	25 (65.8)	0 (0)
	Overweight	13 (23.2)	13 (34.2)	0 (0)
	Obesity	18 (32.2)	0 (0)	18 (100)
Puberal stage, Tanner*	2	33 (58.9)	23 (60)	10 (55.5)
	3	23 (41.1)	15 (39)	8 (44.4)
Leuprolide dose, mg/month*	3.75	48 (85.7)	33 (86.8)	15 (83.3)
	7.5	8 (14.2)	6 (13.2)	3 (16.7)
Leptin, ng/ml		8.30 ± 0.47	7.72± 0.35	9.68 ± 0.77
Adiponectin, mg/dl		6.73 ± 0.23	7.07 ± 0.26	6.01 ± 0.45
Leptin/adiponectin ratio		1.37 ± 0.11	1.15 ± 0.09	1.84 ± 0.26

SE, estándar error; * Frecuency (%).

### Follow-up

During the follow-up, 22 patients (40%) discontinued leuprolide at 24 months of treatment, and 34 patients at 36 months. In all patients, mean BMIz increased throughout follow-up (**Figure 1A**); at the beginning it was 1.22 ± 0.12, at 12 months was 1.35 ± 0.11 (p=0.053), at 24 months 1.39 ± 0.11 (p=0.069), while at the end of leuprolide was 1.46 ± 0.12 (p=0.006), finally, when menarche occurred, between 12 and 18 months after stopping leuprolide, the BMIz was 1.58 ± 0.17 (p=0.157). However, when comparing those with BMI <94 and >95 percentile at diagnosis, it was observed that the first group had an increase in BMIz (mean 0.73 vs. 1.27, *p*=0.01) (**Figure 1B**), contrary to a decrease in the second group (mean 2.26 vs. 2.10; *p*=0.15) (**Figure 1C**).

**Figure 2:**
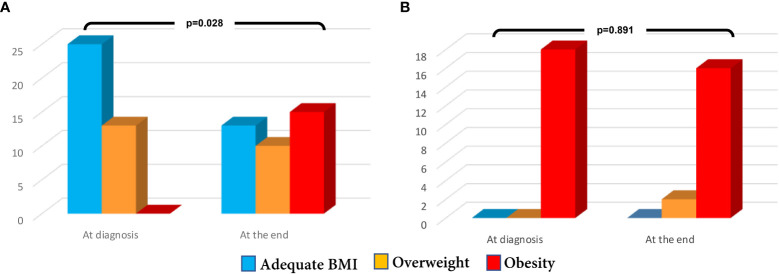
**(A)** Modification of nutrition status in girls with BMI<94 percentile at diagnosis **(B)** Modification of nutrition status in girls with BMI>95 percentile at diagnosis.

Likewise, of the 18 patients who initially had BMI >95^th^ only two patients went from obesity to overweight (**Figure 2B**). While among the 38 patients who started with BMI <94^th^, 21 (55.2%) increase in weight gain; as shown in **Figure 2A**, in the last assessment 15 were classified as obese, 10 as overweight, and 13 as normal weight.

### Adipocytokines

At study onset, the mean leptin value was 8.3± 0.47 ng/ml, and levels were similar according to pubertal developmental stage (Tanner stage II, 8.04 ± 0.60 ng/ml vs Tanner stage III, *p*=0.220), but as shown in **Table 1**, in the BMI <94 group the mean was lower than in the BMI >95 group (p=0.020). And as for adiponectin, the BMI >95 group had lower values than the BMI <94 group, (p=0.020). When analyzing the patients with BMI <94^th^ at baseline, among the 21 patients who had a increase in weight gain, leptin levels were higher than the group of 17 patients who remained unchanged (8.99 ± 0.6 *vs* 6.14 ± 0.8, p=0.005), but the levels of adiponectin were similar between both groups (7.00 ± 0.4 *vs* 7.16 ± 0.3, p=0.61).

**Figure 3:**
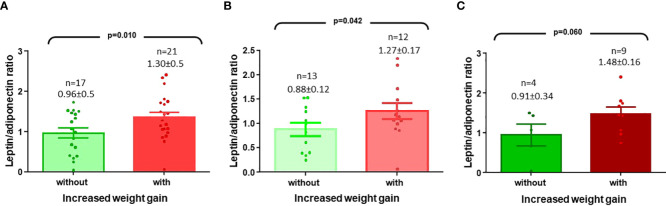
**(A)** Leptin/adiponectin ratio (LAR) values in CCP girls with BMI <94 percentile and who had an increased weight gain **(B)** LAR values in CPP girls with BMI <84 percentile and who had increased in weight gain **(C)** LAR values in CPP girls with BMI >85 and <94 percentile and who had an increased in weight gain.

Regarding LAR at diagnosis, in the 56 patients the mean value was 1.37± 0.11; but in the BMI <94^th^ girls was lower (mean 1.15 ± 0.09), compared to the BMI >95^th^ group (mean 1.84 ± 0.26), p=0.012. As shown in **Figure 3A**, in the 21 patients with increased in weight gain, the values were statistically significantly higher, compared to those of the 17 patients who remained unchanged, 1.30 ± 0.5 *vs* 0.96 ± 0.5, p=0.01. The data were similar when comparing the values in those who started with normal weight (**Figure 3B**) or with overweight (**Figure 3C**), but the LAR mean value was higher in this last group (1.48 ± 0.16).

Finally, multivariate analyses are presented in **Tables 2**, **3** performed on the 38 patients with BMI<94^th^; as shown, both leptin levels and LAR were significantly associated with increased in weight gain, regardless of birthweight, pubertal stage, age, and bone/chronological age ratio; but the strength of association of LAR was greater than leptin levels, RR 1.31, 95%CI 1.03-1.66 *vs* RR 4.86, 95%CI 1.10-21.51, respectively.

**Table 2:** Logistic regression model to determine the association of factors related to the increased weight gain, with leptin and adiponectin levels (n=38).

	RR	CI 95%	P
Leptin, ng/ml	1.31	1.03-1.66	0.026
Adiponectin, mg/dl	0.91	0.57-1.44	0.703
Birth weight, kg	0.99	0.99-1.001	0.908
Puberal stage, Tanner	1.09	0.20-5.85	0.919
Chronological age	0.61	0.32-1.15	0.128
Normal weight and overweight at diagnosis	1.86	0.34-10.09	0.471

RR, Relative Risk; CI, Confidence Interval.

**Table 3:** Logistic regression model to determine the association of factors related to the increased weight gain, with leptin/adiponectin ratio (n=38).

	RR	CI 95%	P
Leptin/adiponectin ratio (LAR)	4.86	1.10-21.51	0.037
Birth weight, kg	0.99	0.99-1.001	0.949
Puberal stage, Tanner	0.74	0.15-3.16	0.327
Chronological age	0.64	0.05-10.58	0.717
Normal weight and overweight at diagnosis	0.77	0.21-2.7	0.690

RR, Relative Risk; CI, Confidence Interval.

## Discussion

Different studies have shown that girls with CPP have a higher risk of weight gain during treatment with GnRHa, as well as having a more adverse cardiometabolic profile, but information on possible associated factors is lacking. To our knowledge, this is the first study to identify LAR as a prognostic marker associated with increased in weight gain in patients with CPP.

Our findings may be relevant, since there is controversy about whether the greater adiposity that CPP patients have at diagnosis may be the main factor related to in the increased cardiovascular risk. In general, these patients tend to increase zBMI, but when analyzed according to their baseline nutritional status, it has been found that CPP girls with normal weight at diagnosis, their zBMI increases more than when they are classified as overweight/obese (8, 9). Park J et al. included 59 patients, of whom 35.6% were overweight or obese; during follow-up until final height was reached, no change in zBMI was observed, but in patients who started overweight/obese, there was a decrease in zBMI (p<0.05) (13). Similarly, in this study, the increase in zBMI was statistically significant in the 38 patients with BMI < 94^th^ (**Figure 1B**), and almost all of those with BMI >95^th^ remained in the same zBMI.

Adipose tissue is metabolically active and secretes adipokines, such as leptin and adiponectin; the former causes vascular inflammation and insulin resistance, while the latter inhibits adherence molecules and increases the production of anti-inflammatory cytokines, such as IL-10 (32–34). In addition, leptin has been shown to be useful prognostic factor in weight gain and in the development of type 2 diabetes mellitus (35, 36). LAR represents a marker of the pathophysiological function of both adipokines and may indicate an imbalance in proinflammatory and anti-inflammatory conditions, as well as adipose tissue dysfunction (37). Under normal conditions, the ratio of leptin and adiponectin is 1:2, which means LAR values of 0.5; thus, higher values have been associated with an increase in cardiovascular risk (21).

To verify the relationship between leptin and adiponectin, studies have been carried out in human white preadipocytes. Singh et al. reported that leptin may regulate adiponectin mRNA *via* extracellular signal-regulated kinase (ERK)-dependent activation of signal transducer and activator of transcription 3 (STAT3), but in obese people these pathways are altered (38).

Adipose tissue dysfunction has been regarded as a form of oxidative stress, in which thiobarbituric acid reactive sub-stances (TBARS) are increased, as well as C-reactive protein (CRP), serum amyloid A (SAA) and osteopontin (OPN) levels (39–42). In adult patients with metabolic syndrome, a positive correlation of LAR levels with CRP has been described, suggesting that adipose tissue dysfunction is related to elevated LAR values (23). This information is consistent with findings from more recent studies in which LAR values could be a more efficient predictor of obesity-related complications compared to leptin or adiponectin levels (41, 43, 44).

LAR values have also been evaluated in children. Some studies in patients without comorbidities with 6-years of follow-up have reported that high leptin levels and LAR are predictors in nonobese subjects. However, it is controversial which of the two is a better predictor; Zhang et al, identified leptin as being better than LAR in change of zBMI (leptin β=0.209 and LAR β=0.146), while Li et al, observed that LAR was better than leptin in change of body fat percentage (leptin β=0.310 and LAR β=0.420) (45, 46). Among girls with CCP we also found that leptin levels and LAR are associated with increased in weight gain, but LAR seems a better marker (**Tables 2**, **3**). In contrast, Yoo JM et al. reported that neither leptin, adiponectin, nor LAR were predictors of weight gain girls with CPP (47); this difference could be due to a shorter follow-up time, or because the analysis was not limited to patients with greater weight gain, as in the present study.

Therefore, we consider that, at the diagnosis of CPP, leptin levels and LAR can be used as a prognostic marker to identify those girls who are at greater risk of significantly increasing their weight during treatment with GnRHa. This would help to offer early interventions to reduce the probability of developing comorbidities related to obesity in adulthood, such as metabolic syndrome, polycystic ovarian syndrome, and type 2 diabetes (48, 49).

As for the limitations of the study, we should mention that the sample size was small, so it seems necessary to carry out more studies to verify our findings. Likewise, in other studies, an attempt should be made to establish the LAR cut-off point that identifies patients with a higher risk of increasing their zBMI.

## Conclusions

In patients with CPP, higher leptin levels and LAR values appear to be associated with significantly greater weight gain during GhRHa treatment, particularly in girls starting with BMI < 94 percentile.

## Data availability statement

The raw data supporting the conclusions of this article will be made available by the authors, without undue reservation.

## Ethics statement

The studies involving human participants were reviewed and approved by the research protocol by the Hospital de Pediatria National Medical Center XXI Century, Instituto Mexicano del Seguro Social ethics and research committee. Written informed consent to participate in this study was provided by the participants’ legal guardian/next of kin.

## Author contributions

Conceptualization, methodology & formal analysis: MV-K & JZ-C. Investigation: JZ-C, LM-A, LD-S, EG-M, AR-H. Writing, review & editing: MV-K & JZ-C. All authors contributed to the article and approved the submitted version.
